# Mitochondrial dysfunction, UPR^mt^ signaling, and targeted therapy in metastasis tumor

**DOI:** 10.1186/s13578-021-00696-0

**Published:** 2021-10-30

**Authors:** Rajendiran Keerthiga, De-Sheng Pei, Ailing Fu

**Affiliations:** 1 College of Pharmaceutical Sciences, Southwest University, Chongqing, China; 2 School of Public Health and Management, Chongqing Medical University, Chongqing, 400016 China

**Keywords:** Mitochondrial unfolded protein response UPR^mt^, Retrograde signaling, Mitohormesis, Hypoxia-inducible factor (HIF), Integrated stress response (ISR), Cytosolic heat shock response (HSR)

## Abstract

In modern research, mitochondria are considered a more crucial energy plant in cells. Mitochondrial dysfunction, including mitochondrial DNA (mtDNA) mutation and denatured protein accumulation, is a common feature of tumors. The dysfunctional mitochondria reprogram molecular metabolism and allow tumor cells to proliferate in the hostile microenvironment. One of the crucial signaling pathways of the mitochondrial dysfunction activation in the tumor cells is the retrograde signaling of mitochondria-nucleus interaction, mitochondrial unfolded protein response (UPR^mt^), which is initiated by accumulation of denatured protein and excess ROS production. In the process of UPR^mt^, various components are activitated to enhance the mitochondria-nucleus retrograde signaling to promote carcinoma progression, including hypoxia-inducible factor (HIF), activating transcription factor ATF-4, ATF-5, CHOP, AKT, AMPK. The retrograde signaling molecules of overexpression ATF-5, SIRT3, CREB, SOD1, SOD2, early growth response protein 1 (EGR1), ATF2, CCAAT/enhancer-binding protein-d, and CHOP also involved in the process. Targeted blockage of the UPR^mt^ pathway could obviously inhibit tumor proliferation and metastasis. This review indicates the UPR^mt^ pathways and its crucial role in targeted therapy of metastasis tumors.

## Background

Mitochondria are essential cellular organelle accountable for crucial cellular pathways such as ATP generation through oxidative phosphorylation, calcium homeostasis, tricarboxylic acid cycle (TAC), innate immunity production, β-oxidation, proteostasis, lipid synthesis, urea cycle, and nucleotide metabolism [1, 2]. These cellular pathways of various mitochondrial functions are tracked to study the retrograde response to recover the organelle from the stress process. The retrograde responses are responsible for gene transcription and protein synthesis to initiate organelle protection [3]. Mitochondrial dysfunction can produce an aggregation of unfolded proteins when mitochondria are suffered from mtDNA mutation, change in mtDNA number, mitochondrial stress, elevated ROS production, and reduction in mitochondrial number. Henceforth, cells activate a transcriptional response to extend the cell's survival, repair, and rescue the dysfunctional mitochondria. This transcriptional response produced in the mitochondria is specified as mitochondrial unfolded protein response (UPR^mt^). UPR^mt^ is currently considered an effective target for tumor theranostics because it plays a crucial role in tumor proliferation and metastasis [4, 5].

The mitochondrial stress which induces UPR^mt^ is due to reduction of mitochondrial DNA (mtDNA), deterioration of mitochondrial ribosome, increased reactive oxygen species level (ROS), oxidative phosphorylation disorder (OXPHOS), increased glucose utilization [6, 7]. UPR^mt^ contemplates the mitochondrial proteostasis and reacts to the stress produced inside the mitochondria by contemporizing the mitochondrial genome and nuclear genomes to produce quality mitochondrial proteome [8, 9]. The quality mitochondrial proteomes for the organelle's recovery are produced by two elementary classes of proteins (i) chaperones (ii) proteases. The proteins play a crucial role in UPR^mt^ by synchronizing mtDNA and chaperones to deliver a quality proteomic genome. Because of the importance of UPR^mt^ in tumor progression and proliferation, and UPR^mt^ inhibition in tumor theranostics and combined drug therapy, this review is intended to study the specific cellular pathways and mechanisms producing UPR^mt^ in the process of tumor proliferation and metastasis.

### Signal transport mediated by UPR^mt^

**Fig. 1:**
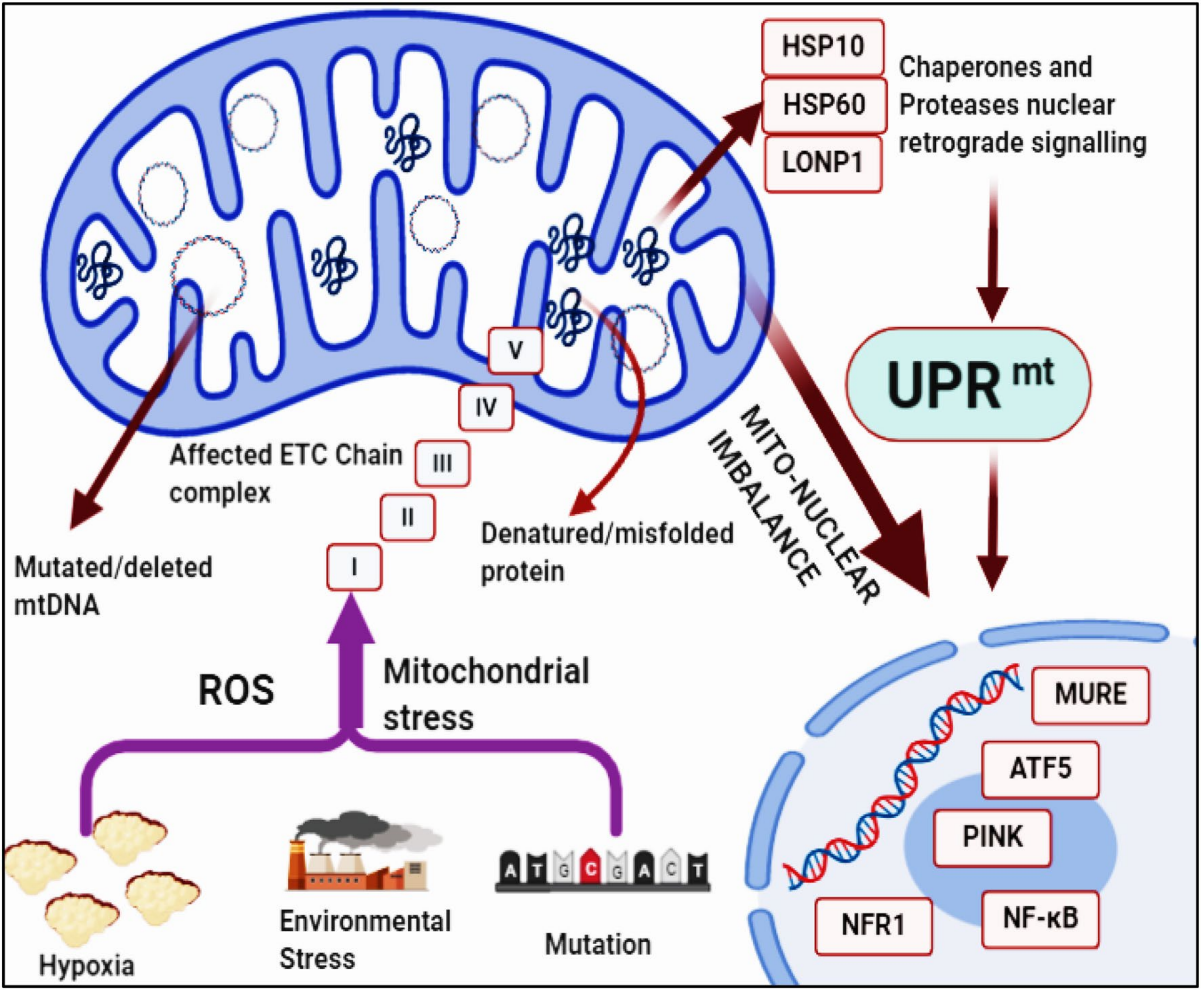
Process of UPR^mt^. The UPR^mt^ signal can be activated by various factors, including hypoxia, evironmental stress, mDNA mutation

The human genomic mitochondria transcribe 22 tRNA, 2 rRNA, and 13 essential proteins, which encodes all four core complexes, namely I, III, IV, and V of the electron transport chain (ETC). Around 99% of the mitochondrial genome proteins of the ETC are transcribed through the nuclear genome. Nuclear DNA encodes the proteome liable for the conservation, replication, and transcription of the mitochondrial genome. For instance, nuclear DNA encoded POLRMT polymerase transcribes the mitochondrial genome [10]. The protein quality control (PQC) network chaperones and proteases on increased mitochondrial proteomic stress induce mitochondria-to-nuclear signaling crosstalk, and one of the crucial factors is UPR^mt^. The UPR^mt^ signal can be activated by various factors, including hypoxia, evironmental stress, mDNA mutation (Fig. 1). Due to its important function in maintaining cell homeostasis, dysregulated UPR^mt^ metabolism leads to the pathogenesis of ischemic diseases, heart diseases, aging, neurogenerative disorders, lung disease, and tumors.

The accumulation of enormous ROS perturbs the mitochondrial stress generated through the ETC. NADH ubiquinone oxidoreductase (complex I) and ubiquinol cytochrome c oxidoreductase (complex III) of ETC directly produce stress by interrupting the structure and the folding mechanism of the proteins [11, 12]. The mitochondrial cytosol heat response (HSR) is induced through the heat shock transcription factor (HSF) by producing cytosolic chaperones, which causes denaturation and misfolding of proteins in mitochondria. Among the two crucial classes of protein Hsp10, Hsp60 and mtHsp70, chaperones and LONP1 proteases hold a critical performance for the UPR^mt^. The mtHsp70 prevents the folded protein's aggregation and is responsible for the translocation of polypeptide through the matrix with associated PAM (Presequence translocase associated motor) subunits [13]. The AAA proteases (ATPase associated cellular activities), namely ClpP and LONP1, degenerate the oxidized and misfolded proteins [14]. Then paralegin (SPG7) and YEML1 arrest the respiratory chain protein misfolding inside the mitochondrial membrane matrix.

### Signal cascade of UPR^mt^ in metastasis tumors

In tumor cells, the mitochondria's activity is dysregulated due to denatured protein, enhancing the prolonged survival and proliferative advantages of tumor cells, causing aggressive malignancies and theranostic resistance [15, 16]. The mitochondrial dysfunction and UPR^mt^ of tumor cells include hypoxia-inducible factor (HIF), proliferative stress, integrated stress response (ISR), and cytosolic heat shock response (HSR) [17]. Also, the mitochondrial biogenetic pathway relies on nuclear DNA (nDNA) and mitochondrial DNA (mtDNA). Mutation of the nDNA and mtDNA produce mitochondrial stress resulting in deregulation of cell signaling and enhanced tumorigenesis, causing impaired respiratory chain function and increased aerobic glycolysis [18, 19].

**Fig. 2:**
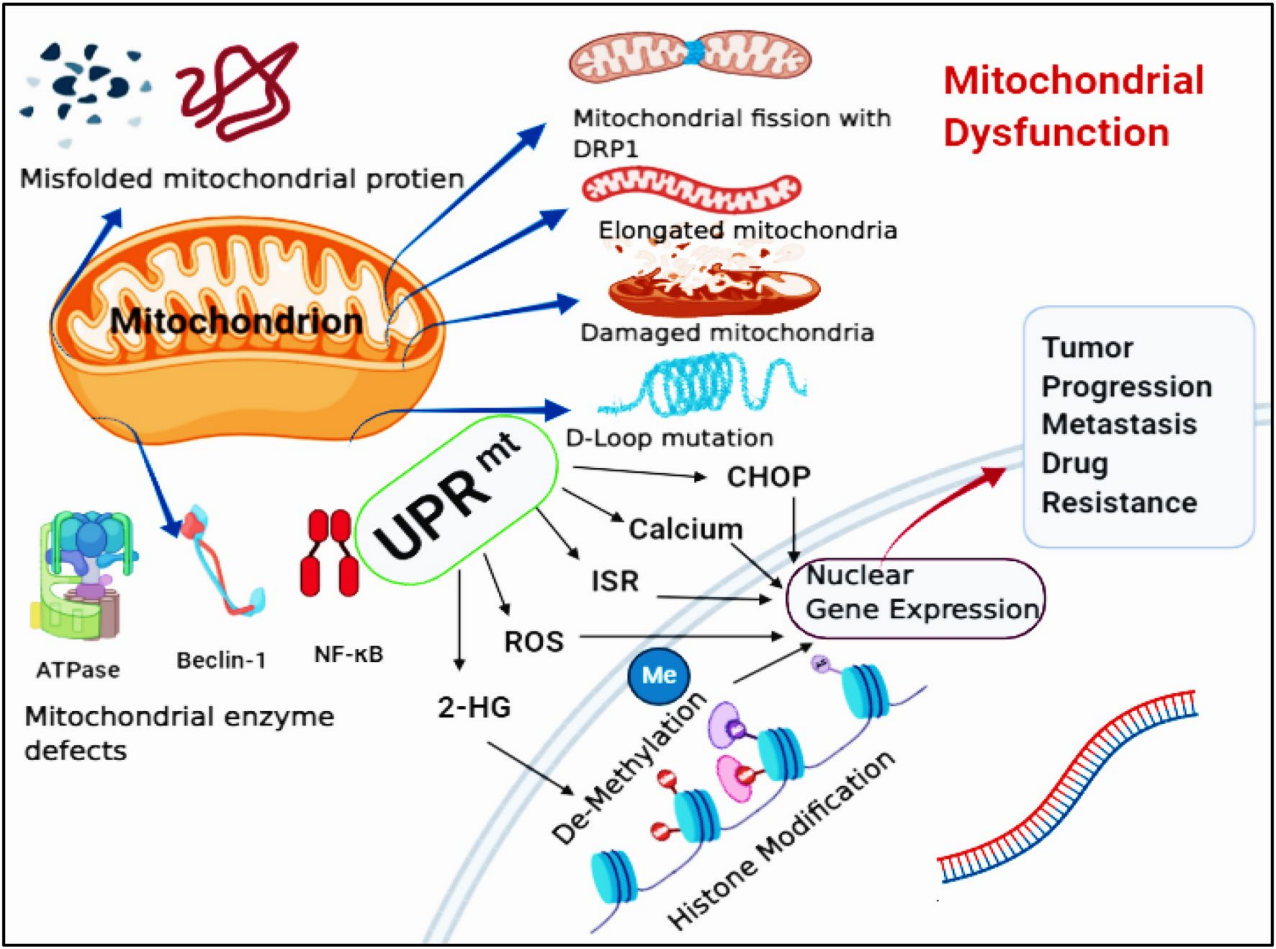
Synopsis of mitochondrial stress response UPR^mt^ inducing tumor progression and tumorigenesis. Stress like mitochondrial damage, altered mtDNA number, mtDNA mutation, mitochondrial enzyme defects, and mitochondrial dysfunction can cause UPR^mt^ and ISR

The quality control, folding process, and import of the mitochondrial proteome and genome are monitored through the UPR^mt^ retrograde transcriptional mechanism. And also, stress like mitochondrial damage, altered mtDNA number, mtDNA mutation, mitochondrial enzyme defects, and mitochondrial dysfunction can cause UPR^mt^, which will induce tumor progression and tumorigenesis (Fig. 2). The UPR^mt^ is transcripted through the expression of mitochondrial chaperones and proteases as a counteraction towards the misfolded protein within the mitochondrial matrix. Based on the endogenous and exogenous stress conditions, tumor cells produce multiple stress response pathways. The cytosolic heat response pathway is one prominent pathway within the cytosol producing chaperones protein, namely HSP27 and HSP90 engaged in protein folding through heat shock factor regulation [20, 21]. The mtDNA mutation enhances mtDNA's depletion, producing overexpression of the nuclear-encoded chaperones such as HSP10 and HSP60 [22, 23]. Thus mitochondrial misfolding and proliferation of the stress protein and aggregates activate UPR^mt^ in tumor cells [24].

Factors such as aging and hypoxia promote mitochondrial misfolding and aggregation of the stress protein within the mitochondrial matrix. The mtDNA is more prone to depletion and mutation due to lack of histones and reduced DNA repair mechanisms within the mitochondria, and also ROS in tumor cells oxidizes the stress protein causing misfolding [25–27]. Differently from normal cells that depend on oxidative phosphorylation for energy supply, carcinoma cells switch to glycolysis for energy production (Warburg effect), which is a hallmark of tumors. Glycolysis regulating factors, such as hypoxia-inducible factor-1 (HIF-1), phosphoinositide 3-kinase/protein kinase B/mammalian target of rapamycin (PI3K/Akt/ mTOR), involve the switch of the glycolytic pathway, contributing to cancer proliferation and metastasis [28, 29]. The rapid proliferation of carcinoma cells further worses the anoxic state and then produces elevated ROS. The prolonged hypoxia induces protein misfolding and accumulation of stress protein, initiating the UPR^mt^ [30, 31]. The import efficacy of activating transcription factors 4 and 5 (ATF 4 and ATF5) is further reduced upon exogenous and endogenous stress [32, 33]. The reduced mitochondrial homeostasis activates the PERK axis of UPR^mt^ and induces the expression of pro-apoptotic protein CHOP, ATF5, and ATF4 [34]. Compared with normal cells, UPR^mt^ exhibit different outcomes in carcinoma cells through multiple signal pathways and effectors, by which UPR^mt^ promotes cell proliferation and metastasis (Table 1).

**Table 1:** Comparison of UPR^mt^ between cancer cells and normal cells

	UPR^mt^ in cancer cells	UPR^mt^ in normal cells
Cell type	All type of the carcinoma cells	Post-mitotic cells
Activitor	Accumulation unfolded proteins, impaired ETC, mtdna mutation and deletion, inhibition of mitochondrial chaperones or proteases, increased ROS level	Accumulation unfolded proteins, impaired ETC, mtDNA mutation and deletion, inhibition of mitochondrial chaperones or proteases, increased ROS level
Regulatory pathway	CHOP-, SIR3/7-, Pink-, Nrf-, calcium-, and ATF4/5-mediated signal pathway	SIR3/7-, and ATF4/5-mediated signal pathway
Effector	FOXOs, HSPs, HIF, ClpP, SOD1/2, MAPK, OXPHOS-related proteins, proteasome, mitochondrial ribosomal protein	HSPs, SOD1/2, OXPHOS-related proteins, proteasome
Outcome	Cancer proliferation and metastasis	Cell longevity and lifespan extension

In order to mitigate the stress, the retrograde signaling of mitochondria to the nuclear genome is activated. The retrograde pathway relies on ROS, ATP production, transcription regulatory components, essential proteins (histone acetylation) [35, 36]. Transcriptional factors of UPR^mt^ such as AKT, AMPK, CHOP identified stress and increased ROS inside the mitochondrial matrix [37]. In C. elegans, during the mitochondrial stress, cytosolic aggregation of ATFS1 encoding both mitochondrial and nuclear signals transcripts UPR^mt^ and OXPHOS genes. The mitochondrial stress recovery is initiated through ATFS-1 action on HSP60, HSP70, and OXPHOS components [38]. In mammalian cells, downregulation of ATF5 retards mitochondrial respiration [33]. And also the proliferation and survival of tumor cells are mediated through gene expression of Egr-1, BCL-2, and MCL1 by ATF-5 [39]. The cell survival and growth of various tumors, namely colorectal, lung tumor, glioma, pancreatic, and breast tumor, are upregulated by ATF5 [40–42]. In addition to the above factors OXPHOS I-V complex impairment also induces UPR^mt^.

In the case of prolonged endogenous mitochondrial stress, the mitochondrial membrane releases cytochrome C inside the cytosol. The cytochrome C reacts with the apoptotic protease activating factor (Apaf-1) to release caspase 9. Thereby apoptosome initiates caspase-9 to activate further caspase-3 and caspase-7, which produces cellular apoptosis [43]. The tumor cells hold an elevated apoptotic threshold than the normal cells resulting in more tumor cell apoptosis [44]. The energy needed for mitochondrial biogenesis is maintained through sirtuins (Sirt 1–7). Sirt 1 binds with NAD^+^ and deacetylates PGC-1α and enhances the transcription and translocation of stress genes HSP60, SOD, and ClpP. The antioxidant mechanism of cells is maintained through polyADP-ribose polymerase (PARP) utilizing NAD^+^. Henceforth the inhibition of the PARP inside the mitochondrial matrix enhances the availability of NAD^+^ for Sirt1. Therefore, Sirt1 promotes the activation of the UPR^mt^ [45]. The nuclear respiratory factor (NRF1) binds with Sirt7 and suppresses mitochondrial metabolism. Further, the reduction in Sirt 7 enhances the stress factor, such as HSP60, HSP10, ClpP, and cell proliferation [46].

UPR^mt^ exhibits high potential stress factors in tumor cells, especially the prosurvival effect of the UPR^mt^ protects the cells from the tumor suppression mechanism. The upregulation of the HSP60, HSP10, SIRT3, and hindrance of CHOP pathway due to UPR^mt^ of the tumor cells enhances the chemoresistance, aggressive growth, and hindered biogenetic pathway inside the tumor cells [47, 48]. The external stress such as hypoxia, mitochondrial DNA mutation, environmental stress affects the electron transport chain causing misfolding and denaturing of the proteins, thereby executing the mito-nuclear imbalance which activates the UPR^mt^ which protects the tumor cells from suppression mechanism and apoptosis.

### Mitochondrial dysfunction, mitochondrial mutation, UPR^mt^, and metastatic tumors

Mitochondrial dysfunction enhanced aerobic glycolysis, and impaired mitochondria are predominantly perceived in tumor cells than in normal cells. In human carcinogenic cells, various mtDNA impairments such as mtDNA copy number variations, mitochondrial enzyme defects, a point mutation in the mitochondria, insertion, and large-scale mitochondrial deletion are widely observed [49]. The mtDNA copy number either increased or decreased in numerous carcinoma, namely in hepatic tumors, gastrointestinal cancer, and breast cancers; the mtDNA copy is reduced. In contrast, the mtDNA copy increases in glioma, lymphoma, colorectal carcinoma, and endometrial adenocarcinoma [50–52]. The predominant mutation in mtDNA is found in the D-loop "hot spot" region in the carcinoma cells [53]. Further, the mtDNA mutation is followed in the protein-encoding region, rRNA, and tRNA genes. The mtDNA mutation subsequently resulted in mitophagy, mitochondrial dysfunction, and increased ROS production [54].

The metastatic mtDNA mutation produces metastasis within non-metastatic nuclei due to enhanced ROS production caused by the *ND6* gene (G13997A and 13885insC) mutation. Ishikawa et al. stated that metastasis is induced through upregulation of nuclear-encoded genes such as *HIF-1a*, *MCL-1*, and *VEGF* [55, 56]. The metastatic breast cancer cell line MDA-MB-231 cells with mitochondrial genome showed complex I defect [56]. Various carcinoma cells exhibit large-scale mtDNA deletion, such as 4977 bp, which inhibit the reduction of 5 tRNA genes and 7 protein-encoding genes. NADPH quinone oxidoreductase 1 (NQO1) deficiency enhances the ROS production in oral and breast cancer due to mtDNA 4977 gene deletion [57–60]. The mtDNA mutations affect the complex I of the electron transport chain in metastatic cancers. The downregulation of NDUFV1 induces complex I dysfunction, which enhances the metastasis [61].

The mitochondrial genome is highly vulnerable to oxidative defects and ROS production due to OXPHOS impairment due to mutations. The ROS production induces an apoptosis signal in the tumorigenesis pathway [62, 63]. McMahon et al. studied breast cancer from 99 women; around 73.7% of women exhibited somatic mtDNA mutation encoding for complex I [64]. Yuan et al. identified nonsense mtDNA mutation in the *ND6* gene of lung adenocarcinoma, inducing increased ROS production [65]. Carcinogenic cell mutations appear in the mitochondrial enzymes such as fumarate hydratase (FH), succinate dehydrogenase (SDH), and isocitrate dehydrogenase (IDH). The FH mutation of mitochondrial enzyme induces enhanced carcinogenic risk in renal carcinoma and leiomyosarcoma, SDH mutation induces carcinoma in neuroblastoma, and IDH causes malignant cancers like glioma, myeloid neoplasia, chondrosarcoma, and cholangiocarcinoma [66–68]. Mitochondrial nicotinamide adenine dinucleotide-dependent deacetylase, Sirtuin-3 (Sirt 3) defect downregulates mtDNA repair gene (8-oxoguanine DNA glycosylase, OGG1-2a), increasing the proliferation of oral cancer, breast cancer, head and neck carcinoma [69, 70].

Mitophagy plays an important role in mitochondrial quality control and cell survival through selective removal of dysfunctional or damaged mitochondria. In normal cells, mitophagy prevents the accumulation of the damaged organelles and inhibits cell carcinogenesis by maintaining a pool of healthy mitochondria. However, mitophagy can provide nutrients for cancer cells by degrading organelles and then promotes tumor growth, since the mitophagy regulators of cancer cells comprise a various of constituents that regulate stress response, cell cycle, survival pathway and ECM detachment during carcinoma proliferation and metastasis, such as AMPK, FOXOs, Sirtuins, ATF4/5 [71]. Therefore, mitophagy can be used as an anticancer target to inhibit cancer cell proliferation.

The endogenous and exogenous stress causes mitochondrial dysfunction, which further exhibit retrograde signalling to regulate the cellular homeostasis and protect the cells through retrograde regulation of genes. The mitochondrial subunits, such as mtDNA, mtRNA, human, and MOTS-c, hold a crucial role in retrograde signalling [72]. The increased ROS production in cancer initiates the retrograde signalling to enhance antioxidant activity through nuclear erythroid related factors 2 (NRF2), enhancing mitochondrial biogenesis through the JNK-PGC1a pathway and increase mitochondrial complex II phosphorylation [73–75]. Also, in tumor cells, the increased ROS enhances tumor progression through nuclear factor-jB (NFjB). The mtDNA mutation, mitochondrial dysfunction, and defective OXPHOS can induce Ca^+^ release from mitochondria. The cytosolic calcium in the mitochondria induces calcium retrograde signaling via activation of NF-jB, Jun-N-terminal kinase (JNK) and p38 MAPK pathway, upregulation of CREB, early growth response protein 1 (EGR1), ATF2, CCAAT/enhancer-binding protein-d and CHOP [76, 77]. Thus ROS and Ca^2+^ play a crucial role in the mitochondrial mechanism.

**Fig. 3:**
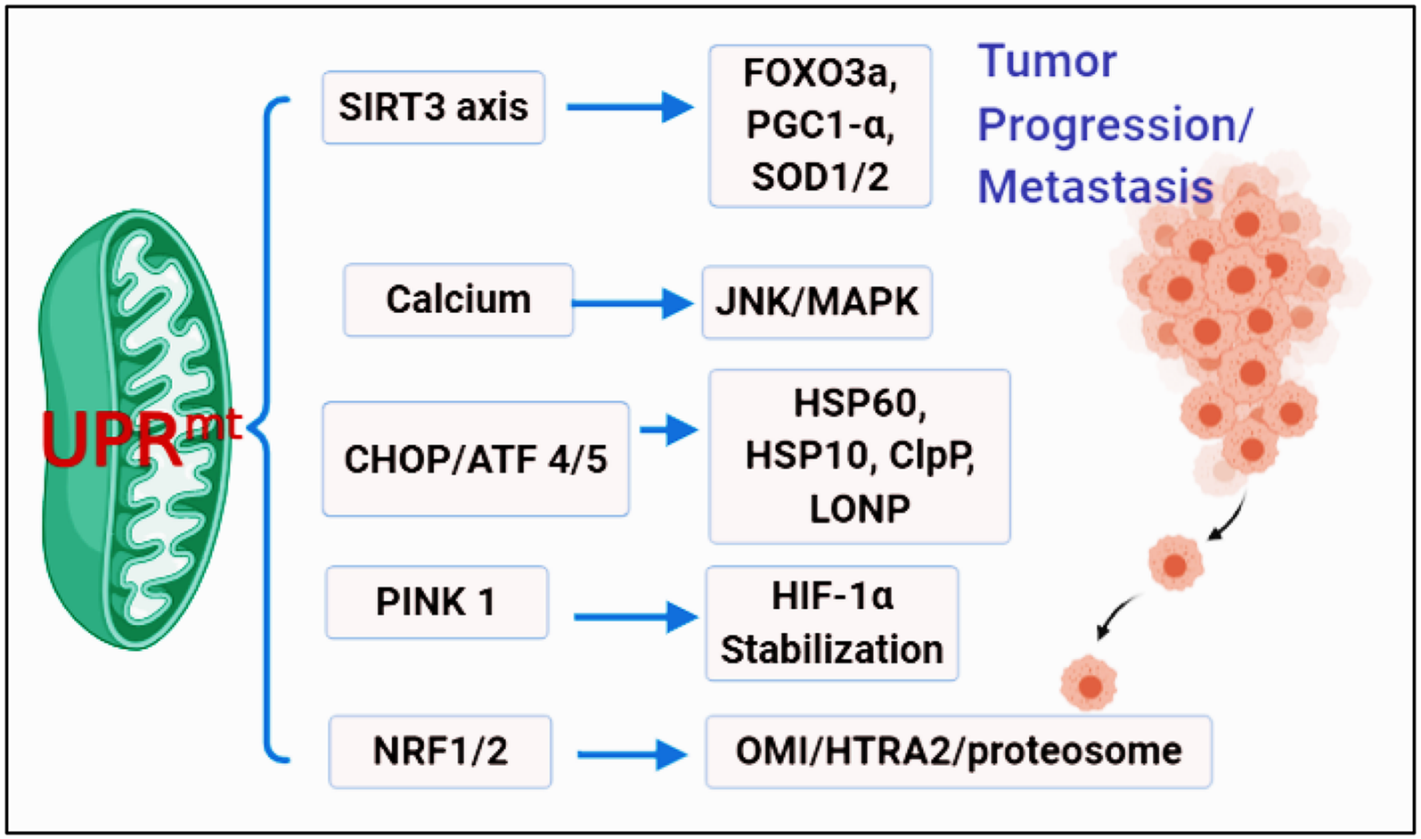
UPR^mt^ is activated through different axis, leading to metastasis

In UPR^mt^, the upregulated mitochondrial misfolded ornithine transcarbamylase (OTC∆) activates the transcription of CHOP, proteases, ClpP, and chaperones HSP60, HSP70 due to the proteomic mitochondrial stress. The OMI/HTRA2, NRF1, and proteosome transcription are activated through the estrogen receptor alpha (Erα) of the UPR^mt^ axis, and the SIRT3 UPR^mt^ axis induces antioxidant genes and helps in the removal of damaged mitochondria through mitophagy [78, 79]. In ISR, electron transport chain (ETC) dysfunction, increased ROS, and UPR^mt^ induce GCN2, PERK, and HRI based on the stress of the tumor environment. In UPR^mt^, integrated stress response (ISR) plays a key role in adaptation to stress. The ISR acts based on eukaryotic translation initiation factor 2α kinases (eIF2α) accountable for cap-dependent protein translation and activation transcription factor-4 (ATF-4) [80]. The eIF2α- ATF-4 pathway is more prominent in the tumor cells. The downregulation of ATF4 decreases the carcinoma, whereas the upregulation of ATF4 promotes tumor progression through GCN2 activation [81, 82]. The UPR^mt^ axis, including SIRT3, PERK, CHOP, ATF4/5, ETC pathways, mainly aggravates the tumor progression (Fig. 3, Table 2).

Upregulation of HSP60 and ATF5 during UPR^mt^ predominantly increases the carcinoma cell’s survival threshold and promotes tumor progression, therapeutic resistance, and resistance towards apoptosis [39, 83]. Inhibition of ATF5 enhances the growth of human glioblastoma cells and human pancreatic cancer cells [84]. The UPR^mt^ relies on SIRT3 for deacetylation and further relocalization of FOXO3a to the nucleus and SOD2 for the antioxidant response. The significant increase in SIRT3/FOXO3a/SOD2 UPR^mt^ axis demonstrated a high risk of tumor progression in head and neck cancer [85]. UPR^mt^ in acute myeloid leukemia showed increased cell apoptosis due to BCL2 inhibition caused by knockdown of SDHB, thereby affecting the respiratory chain complex II [86]. In ROS production and UPR^mt^ superoxide dismutase, SOD1 and SOD2 play a vital role. On UPR^mt^ activation, the intermembrane space (IMS) found SOD1 found to be increased than SOD2 in breast carcinoma cells [10, 87].

The UPR^mt^ activation of breast cancer exhibits depletion of mitochondrial metallopeptidase OMA1, causing mitochondrial homeostasis and increased ki67 expression genes promoting metastatic growth of cancer [88]. The inhibition of ClpP exhibit the anti-tumor effect in both in vitro and in vivo conditions in the leukemia cells. The upregulation of ClpP is prominently found in metastatic tumors [89, 90]. In UPR^mt^ activated breast carcinoma cells, overexpression of Her-2 is observed by Chen et al. [91]. The UPR^mt^ exhibits initiation of c-jun through binding of JNK2 to increase CHOP and C/EBPβ, which increase HSP60 and HSP10 in breast cancer [34]. Further mtDNA mutation targeting ND3 (A10398G) in bone carcinoma cells colonizes bone carcinoma cells [92]. The breast carcinoma cells contain low SIRT3 than the normal MCF10A cells due to UPR^mt^. SIRT3 and SOD2 are decreased in the breast carcinoma cells with activated UPR^mt^ [93]. Hu et al. stated that the carcinoma cells upregulate genome BNIP3, a mitophagy enhancing agent, to adapt to hypoxia produced during antiangiogenic theranostics [94]. The enhanced ROS level in the carcinoma cell promotes increased metastasis and invasiveness due to enhanced UPR^mt^ to maintain cell toxicity and cell viability [95].

Further, Lin et al. observed elevated fascin level through the enhanced oxidative mechanism in lung carcinoma cells in the metastatic stages due to mitochondrial F-actin's stability [96]. The knockdown of BRCA1 or BRCA2 gene expression in breast carcinoma cells increases hydrogen peroxide formation in carcinoma cells and neighboring stroma cells [97, 98]. The knockdown of BNIP3 expression is prominently found in pancreatic and breast cancer progression [99–102]. Upregulation of carbonic anhydrase IX (CAIX) is induced through UPR^mt^ through transcription of HIF-1α, thereby increasing high-risk carcinoma proliferation, metastasis, and locoregional failure [103, 104]. The upregulation of SIRT3 due to UPR^mt^ elevates the ROS production and stabilization of HIF-1α, which initiates the switching of the anaerobic glycolytic process, the Warburg effect in various carcinomas, including breast cancer, hepatic, gastric, and colorectal carcinoma [105].

The switching of the anaerobic glycolytic process retard/delete the Parkin or Pink1, thereby increasing ROS and HIF-1α deletion, enhancing tumorigenesis and proliferation of kras-mutant pancreatic ductal adenocarcinoma (PDAC) [106]. NIX (BNIP3L) expression is responsive for UPR^mt^, and it downregulates sphingosine kinase 1 (SPHK1) localized in mitochondria [107, 108]. The glycolytic process increases the mitochondrial Ca^2+^ through mitochondrial calcium uniporter (MCU), thus overexpressed MCU channels are widely found in breast carcinoma patients [109, 110]. The pyruvate dehydrogenase kinase 1 (PDK1) controls the mitochondrial quality and plays a crucial role in the TAC cycle and OXPHOS I-V complex. The downregulation of PDK1 initiates mitochondrial quality disorders and increases metastasis [111]. Sun et al. observed increased mitochondrial fission and upregulation of Drp1 expression in the hepatocellular carcinoma cells, and further, it promotes proliferation and metastasis [112].

In various types of carcinomas, overexpression of mitochondrial ribosomal protein (MRPs) such as MRPL38, MRPS27, and MRPL10 are widely observed due to transcription of UPR^mt^ [113]. The knockdown of SIRT3 expression in tumor growth increases ROS production and focal adhesion kinase (FAK) activation [114]. Mitochondrial transcriptional factor A (TFAM) mediate and regulate the mtDNA copy number, defective mitochondria, damaged molecular pattern, inflammation. TFAM initiates the mtDNA copy number through enhanced OXPHOS in colorectal carcinoma. Further, TFAM affects the calcium transport, flagella associated protein 65(CFAP65) synthesis, and cytoplasmic phosphoenolpyruvate carboxykinase (PCK1) expression through retrograde mitochondrial signaling and UPR^mt^ activation, which further increases carcinoma proliferation and progression [115, 116]. Mitohormesis is observed due to UPR^mt^ activation, which produces carcinoma invasion, multiplication, and metastasis in various cancers [95]. FH and SDH mutations initiate retrograde mitochondrial signaling, leading to the accumulation of fumarate and succinate in the carcinoma cells due to UPR^mt^. Further accumulation of fumarate, succinate, 2-HG (D-2-hydroxyglutaric acid) enhances malignancies. And also, 2-HG affects the metabolism of complex IV/V resulting in deregulation of the mitochondrial energetics, stabilization of HIF-1α, and carcinoma progression. Further 2-HG accumulation produces mtDNA de-methylation causing genetic mutation in the carcinoma cells [117–119]. The retrograde signaling initiates the loss of heterozygosity (LOH) observed in the carcinoma patients' leiomyomatosis and renal carcinomas due to germline FH mutations [120]. The mitochondrial dysfunction and retrograde mitochondrial signaling evidentially produced overexpression of fibroblast growth factor 21 (fgf21) and growth differentiation factor 15 (gdf15) in tumor patients [121, 122].

The UPR^mt^ through exogenous and endogenous stress alters the epigenome through substantial chromatin reorientation initiated through histone, namely, methyltransferase MET 2 and nuclear cofactor LIN65, exhibiting switching in the pattern of H3K9me methylation. During mitochondrial dysfunction initiated chromatin alteration and downregulation of the UPR^mt^ genes, the transcriptional regulators ATFS 1 and DVE 1 initiates proteostasis and cell longevity [123, 124]. The further studies on UPR^mt^ explained that the transcriptional genes activating UPR^mt^ contain two supplementary elements on both sides of the CHOP/CEBPβ component called mitochondrial response elements (MURE 1 & 2) [125]. The minor missense mutation of the mitochondrial genome in the non-protein region promotes metastasis in the carcinoma cells [126]. The mutated mitochondrial DNA further affects and retard the mtDNA copy number due to activated UPR^mt^, further enhancing mitochondrial biogenesis and mtDNA replication [127]. The studies revealed mtDNA alterations and mtDNA mutations play a crucial role in the activation of UPR^mt^, which in turn increases mitochondrial health, which further positively influences the proliferation and metastasis in the carcinoma [128]. The synopsis of the mitochondrial dysfunction, UPR^mt^ metabolism, and pathway are precisely tabulated as follows (Table 1).

### Tumor theranostics and UPR^mt^

**Fig. 4:**
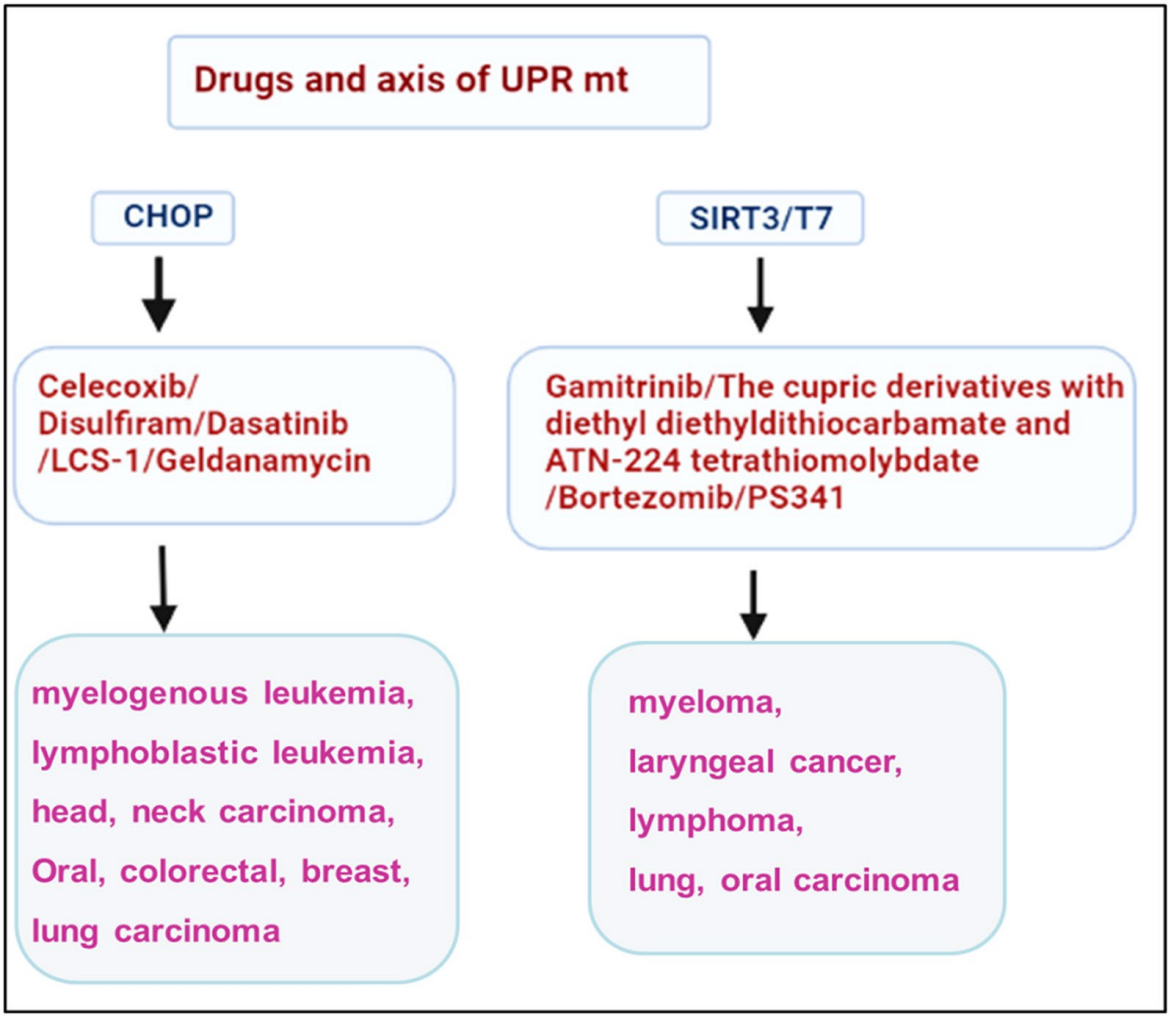
Schematic representation of drugs targeted to UPR^mt^ axis

Mitochondria-nuclear retrograde pathway (mito-nuclear pathway) is a signal communication from mitochondria to nucleus. Mito-nuclear pathway employs various retrograde signals to regulate nuclear gene expression to maintain cell homeostasis. UPR^mt^ is an important pathway in the retrograde mito-nuclear communication widely observed in carcinoma cells. The activated UPR^mt^ pathway can induce nuclear gene to express various proteins to stabilize the structure of dysfunctional mitochondria of carcinoma cells, which will continue to provide metabolic intermediates for maintaining the cell proliferation. Henceforth UPR^mt^ can be utilized as a specific target for drugs to inhibit tumor growth (Fig. 4). Selective drugs target the inhibition of proteases, and chaperones should be synthesized to produce almost 100% efficacy in the treatment of carcinoma. The anti-tumor drugs such as Bortezomib and nelfinavir, which are proteasome inhibitors, are utilized to hinder the UPR pathway through downregulation of VEGF factor to retard the tumor vasculature [129–134]. Geldanamycin inhibits the UPR pathway's chaperones, reduces the HIF1-α stabilization, and stimulates the hypoxic carcinoma death [135]. The tumor theranostic utilizing Gamitrinib enhanced specific tumor apoptosis through inhibiting TNF receptor-associated protein-1 (TRAP-1) chaperones [136, 137]. The therapeutic anti-cancer agent LCS-1 inhibited the progression of lung carcinoma cell, neck, head carcinoma [138, 139]. The cupric derivatives with diethyl diethyldithiocarbamate and ATN-224 tetrathiomolybdate inhibited the SOD1 pathway of the tumor progression and thereby exhibited apoptosis of lung carcinoma [140, 141]. The carcinoma prodrug of glutamine antagonist 6-diazo-5-oxo-l-norlecuine (DON) improved the T cell mitochondrial metabolism in the tumor cells to enhance the efficacy of the anti-tumor effect [142]. Bortezomib/PS341 is one of the potential di-peptidyl boronic acid components utilized as the 26S proteasome inhibition in treating myeloma, laryngeal cancer, and lymphoma [143]. Moreover, the drugs that target UPR^mt^ include Carfilzomib and Oprozomib, which are widely utilized in oral, HNSCC, and multiple myeloma in cancer therapy by inhibiting MCl-1 [144]. The UPR^mt^ activation of disulfiram induces apoptosis in oral and pharyngeal tumor cells via CHOP [145]. Celecoxib drug studies illustrated the ant-cancer activity and induced apoptosis in oral, head, neck, colorectal carcinoma via CHOP and BNIP3 pathway [146]. The pyrimidine and thiazole based drug dasatinib studies revealed effective anti-cancer activity in myelogenous leukemia, lymphoblastic leukemia, head and neck carcinoma. Dasatinib inhibits cancers via knockdown of AMPK and CHOP pathways of carcinoma. Dasatinib is a multikinase inhibitor that has been approved by FDA for treating chronic myelogenous leukemia. Nevertheless, the anticancer machanism of dasatinib is more complex than expected. For example, AMPK-dependent stress is proved to involve the dasatinib-induced apoptosis [147]. In addition, the inhibition effect of dasatinib on gastric cancer is reportd that is mediated by CHOP [148].

Moreover, mitochondrial therapy (mitotherapy) are found to be one efficient methodology to treat carcinoma cells. Mitotherapy is to transfer the isolated healthy mitochondria into culured cells by incubation and into animals by injection, then the exogenous mitochondria will play roles in cells. Recent studies have shown that the mitotherapy can inhibit rapid proliferation of tumors, including breast cancer, glioma, and melanoma. Studies have revealed that mitotherapy can inhibit tumor cell glycolysis, and reduce ATP and lactate production after the healthy mitochondria enter cancer cells. In addition, healthy mitochondria can reduce oxidative damage, thereby retards UPR^mt^ and cancer proliferation [149–152]. In addition, efficient anti-cancer drug and cancer apoptosis can be achieved through targeting the mitochondrial stress response components like ClpP, proteases, and chaperones [89, 153–157]. The various FDA approved UPR^mt^ components, chaperones, and proteasome inhibitor drugs of carcinoma are listed below in Table 3.

**Table 2:** Mitochondrial dysfunction and pathway of UPR^mt^ in carcinoma cells

Carcinoma cells	Mechanism of UPR^mt^ activation	Type of axis of activation	References
Lung, breast, glioma cells	HSP60, HSP10 stress protein	CHOP	[23, 24]
Glioblastoma, melanoma, prostate carcinoma cells	Gene expression of Egr-1, BCL-2, and MCL1	CHOP/ATF	[39]
Lung, Pancreatic, Breast, Glioma	ATF-5	CHOP	[40–42]
Breast cancer	PARP inhibition, NRF1 with SIRT7	CHOP/SIRT3	[45, 46]
Hepatic, gastrointestinal, breast carcinoma	Alteration in the mtDNA copy	CHOP/SIRT3/PINK	[49–52]
Breast Carcinoma	ROS production through ND6 gene mutation, upregulation of VEGF	PINK/JNK/HIF-α stabilization	[55]
Breast cancer	Defect in mitochondrial gene complex I	SIRT3/CHOP	[56]
Oral, breast cancer	NQO1 deficiency,4977 gene deletion	SOD/NRF1	[57–60]
Metastatic cells	Downregulation of NDUFV1	NRF1	[61]
Breast cancer	mtDNA mutation	CHOP/SOD/NRF1	[64]
Lung adenocarcinoma	mtDNA ND6 gene mutation	CHOP/SOD/NRF1	[65]
Renal, neuroblastoma, glioma	FH, SDH, IDH enzyme mutation	CHOP/NRF1	[66–68]
Head, neck, oral, breast	Downregulation of OGG1-2a	SIRT3	[69, 70]
Breast, Renal and Pancreatic carcinoma	PINK1/BCl-2, BNIp3/NIX overexpression	SIRT3/CHOP/NRF1	[71]
Hepatocellular carcinoma cells	ROS production	NRF2/PGC 1a/JNK	[73–75]
Fibrosarcoma (mesenchymal tumor)	Calcium dysfunction, Inhibition of NF-κB, Ros production	CHOP/EGR1/JNK/MAPK	[76, 77]
Breast carcinoma cells	OMI/HTRA2,NRF1	SIRT3	[78, 79]
Fibrosarcoma, colorectal adenocarcinoma	Knockdown of ATF4, ATF4 expression inhibition through GCN2 activation	CHOP/SIRT3	[81, 82]
Glioblastoma, pancreatic cancer	Inhibition of ATF5	CHOP	[84]
Head, neck cancer	SIRT3/FOXO3a/SOD2	CHOP/SIRT3	[85]
Myeloid leukemia	Inhibition of Bcl2, knockdown of SDHB	NFR1/2	[86]
Breast cancer	ROS production	NFR1/2/CHOP	[10, 87]
Breast cancer	Depletion of OMA1 increased gene expression ki67	CHOP/NFR1/PINK	[88]
Myeloid leukemia metastatic cancer	Inhibition of ClpP	CHOP	[89, 90]
Breast cancer	Overexpression of Her2	CHOP	[91]
Breast cancer	Inhibition of JNK2	CHOP	[34]
Bone carcinoma	ND3 mutation	CHOP/SIRT3	[92]
Breast carcinoma	ROS production, SOD1/2	SIRT3	[93]
Glioblastoma	BNIP3 upregulation	CHOP/SIRT3/NFR1/2	[94]
Lung carcinoma	Elevated fascin	CHOP	[96]
Breast	Knockdown of BRCA1/2	SOD/NFR1	[97, 98]
Glioblastoma	Downregulation of PINK, HIF-1α stabilization	CHOP/PINK/NRF	[99]
Breast, pancreatic cancer	Knockdown of BNIP3	CHOP/NRF1/SOD	[100–102]
Breast, neck, colorectal, head carcinoma	Upregulation CAIX, HIF 1-α stabilization	CHOP/SIRT3	[103, 104]
Gastric, breast, colorectal carcinoma	ROS production, HIF 1-α stabilization	SIRT3	[105]
Pancreatic ductal adenocarcinoma	ROS production, HIF 1-α deletion	PINK1/Parkin	[106]
Ovarian, lung, colorectal carcinoma cells	BNIP3/NIX downregulates SPHK1	CHOP/SIRT3	[107, 108]
Breast cancer	Overexpression of MCU	Calcium/CHOP	[109, 110]
Liver cancer lining	Knockdown of PDK1	CHOP/NRF1/2	[111]
Hepatocellular carcinoma	Upregulation of DRp1, mitochondrial fission	CHOP/NRF1	[112]
Renal carcinoma cells	2-HG, de-methylation of histone	CHOP/PINK	[117–119]
Renal and colorectal carcinoma cells	Overexpression of FGF21, GDF15	CHOP/NFR1	[121, 122]

**Table 3:** Drugs inhibiting UPR^mt^-mediated chaperones and proteases pathway in carcinoma cells

Drug	Carcinoma type	Inhibition pathway	References
Geldanamycin	Metastatic cells, breast, lung carcinoma	Inhibition of CHOP/HIF1-α	[135]
Gamitrinib	Oral, breast, hepatocellular	Inhibiting sirt3/7-tnf receptor-associated protein-1 (trap-1)	[136, 137]
LCS-1	Lung carcinoma cell, neck, head carcinoma	Inhibition of chop	[138, 139]
The cupric derivatives with diethyl diethyldithiocarbamate and ATN-224 tetrathiomolybdate	Lung carcinoma cell	Inhibited the sirt7/sod1 pathway	[140, 141]
Bortezomib/PS341	Myeloma, laryngeal cancer, and lymphoma	Inhibition of sirt7/26 s proteasome	[143]
Disulfiram	Oral and pharyngeal tumor cells	CHOP	[145]
Celecoxib	Oral, head, neck, colorectal carcinoma	CHOP/BNIP3	[146]
Dasatinib	Myelogenous leukemia, lymphoblastic leukemia, head and neck carcinoma	Inhibition of CHOP and AMPK pathway	[147, 148]

## Conclusion

The oncology and tumor therapeutic field in realizing that mitochondrial metabolism plays a crucial role in modeling the futuristic drug would achieve great progress. Mitochondrial dysfunction, including change in mtDNA copy number, mtDNA mutation, mitochondrial enzyme defects activate the UPR^mt^ retrograde signal from mitochondria to nucleus, then nuclear genes express mitochondria-related proteins to protect the dysfunctional mitochondria, and meanwhile to facilitate the dysfunctional mitochondria to provide energy and intermediate metabolites for tumor proliferation and metastasis. The critical importance of cancer modeling therapeutic should target the UPR^mt^ through small molecule drug therapy and mitotherapy.

**Fig. 5:**
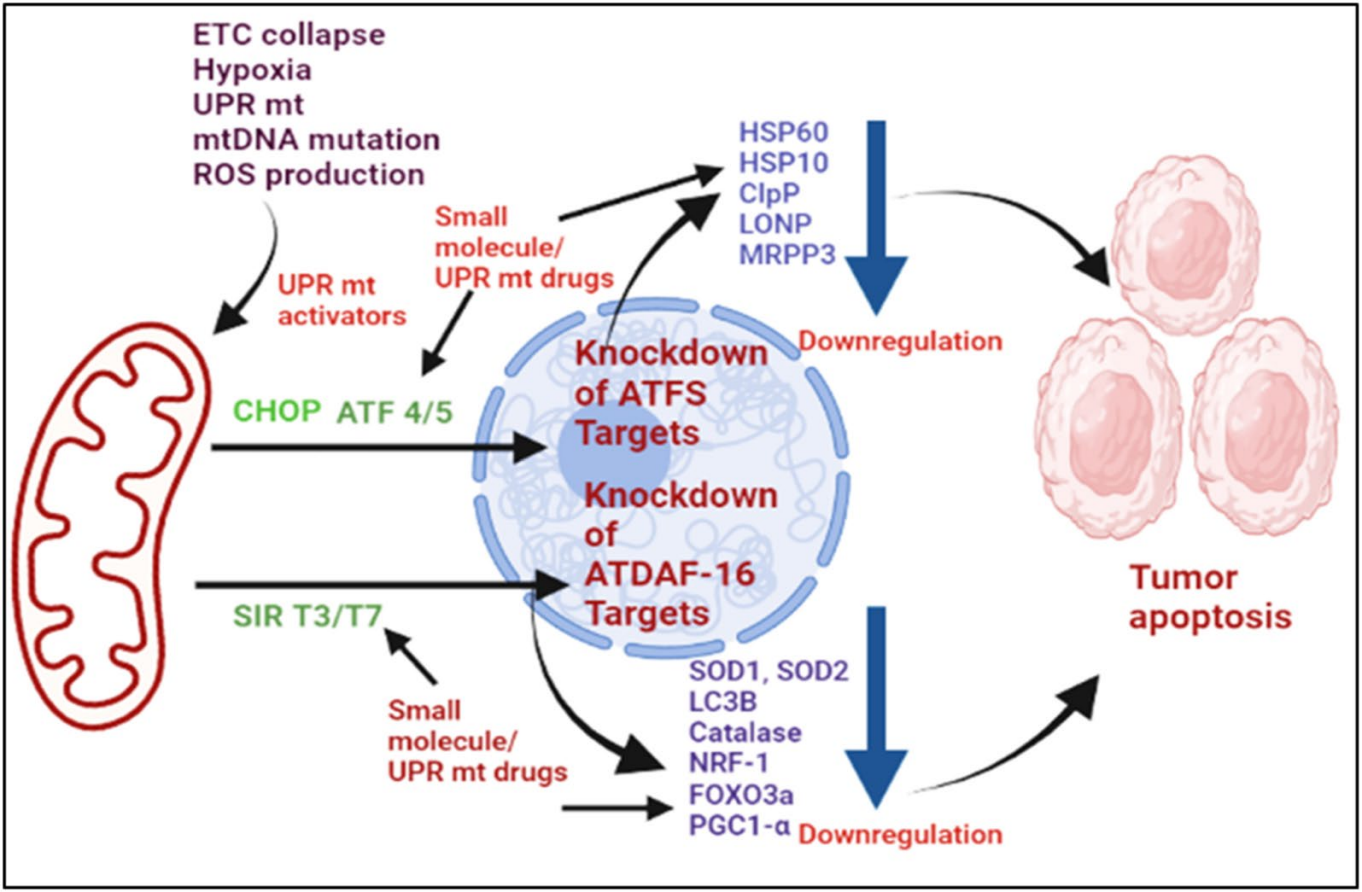
Downregulation of CHOP, ATF and SIR signal of cancer cell through small molecule drugs

On the basis of understanding the molecular mechanism of UPR^mt^, targeted downregulation of UPR^mt^ signal molecules, including CHOP, ATF-5, and SIRT3, would retard tumor growth and induce the cell apoptosis (Fig. 5). And also, drugs that target the CHOP/SIRT3/NRF1/2 signal pathway should achieve maximum tumor death or eradication efficacy. Therefore, the exploitation of targeted drugs for blocking UPR^mt^ is a prominent strategy to treat metastasis tumors through a sustainable mechanism in tumor therapy.

## Data Availability

Not applicable.

## Abbreviations

AKT: Serine/threonine-protein kinase
AMPK: Adenosine 5’-monophosphate (AMP)-activated protein kinase
Apaf-1: Apoptotic protease activating factor
ATF: Activating transcription factor
BNIP3: BCL-2/adenovirus E1B protein-binding protein 3
CFAP65: Cilia and flagella associated protein 65
CHOP: C/EBP homologous protein
ClpP: Caseinolytic peptidase
CREB: CAMP-response element binding protein
DON: 6-Diazo-5-oxo-L-norlecuine
EGR: Early growth response protein
EGR1: Early growth response protein 1
eIF2α: Elongation Initiation factor 2α kinases
Erα: Estrogen receptor alpha
ETC: Electron transport chain
FAK: Focal adhesion kinase
fgf21: Fibroblast growth factor 21
FH: Fumarate hydratase
gdf15: Growth differentiation factor 15
HIF: Hypoxia-inducible factor
HSF: Heat shock transcription factor
HSP: Heat shock protein
HSR: Cytosolic heat shock response
IDH: Isocitrate dehydrogenase
ISR: Integrated stress response
JNK: Jun-N-terminal kinase
LONP: Lon protease
MCU: Mitochondrial calcium uniporter
MRP: Mitochondrial ribosomal protein
mtDNA: Mitochondrial DNA
NADH: Nicotinamide adenine dinucleotide
NADPH: Nicotinamide adenine dinucleotide phosphate
NQO1: Quinone oxidoreductase 1
NRF: Nuclear respiratory factor
NRF2: Nuclear erythroid related factors 2
OGG1-2a: 8-Oxoguanine DNA glycosylase
OXPHOS: Oxidative phosphorylation disorder
PARP: PolyADP-ribose polymerase
PCK: Cytoplasmic phosphoenolpyruvate carboxykinase
PDK: Pyruvate dehydrogenase kinase
PINK1: PTEN-induced putative kinase 1
PQC: Protein quality control
ROS: Reactive oxygen species
SDH: Succinate dehydrogenase
SIRT: Sirtuin
SOD: Superoxide dismutase
TAC: Tricarboxylic acid cycle
TRAP-1: TNF receptor-associated protein-1
UPR^mt^: Mitochondrial unfolded protein response

## References

[1] Wouters BG, Koritzinsky M (2008). Hypoxia signalling through mTOR and the unfolded protein response in cancer. Nat Rev Cancer.

[2] Pellegrino MW, Nargund AM, Haynes CM (2013). Signaling the mitochondrial unfolded protein response. Biochim Biophys Acta Mol Cell Res.

[3] Münch C, Harper JW (2016). Mitochondrial unfolded protein response controls matrix pre-RNA processing and translation. Nature.

[4] Qureshi MA, Haynes CM, Pellegrino MW (2017). The mitochondrial unfolded protein response: signaling from the powerhouse. J Biol Chem.

[5] Nagelkerke A (2014). The unfolded protein response as a target for cancer therapy. Biochim Biophys Acta Rev Cancer.

[6] Kim R (2006). Role of the unfolded protein response in cell death. Apoptosis.

[7] Mohamed E (2020). The unfolded protein response mediator perk governs myeloid cell-driven immunosuppression in tumors through inhibition of STING signaling. Immunity.

[8] Cole DW (2019). Targeting the unfolded protein response in head and neck and oral cavity cancers. Exp Cell Res.

[9] Jovaisaite V, Mouchiroud L, Auwerx J (2014). The mitochondrial unfolded protein response, a conserved stress response pathway with implications in health and disease. J Exp Biol.

[10] Kenny TC, Gomez ML, Germain D (2019). Mitohormesis, UPRmt, and the complexity of mitochondrial DNA landscapes in cancer. Cancer Res.

[11] Zhao RZ (2019). Mitochondrial electron transport chain, ROS generation and uncoupling. Int J Mol Med.

[12] Hamanaka RB, Chandel NS (2010). Mitochondrial reactive oxygen species regulate cellular signaling and dictate biological outcomes. Trends Biochem Sci.

[13] Liu Q, Liang C, Zhou L (2020). Structural and functional analysis of the Hsp70/Hsp40 chaperone system. Protein Sci.

[14] Hamon MP (2020). Mitochondrial Lon protease-depleted HeLa cells exhibit proteome modifications related to protein quality control, stress response and energy metabolism. Free Radic Biol Med.

[15] Ward PS, Thompson CB (2012). Metabolic reprogramming: a cancer hallmark even warburg did not anticipate. Cancer Cell.

[16] Hanahan D, Weinberg RA (2011). Hallmarks of cancer: the next generation. Cell.

[17] Kreuzaler P (2020). Adapt and conquer: metabolic flexibility in cancer growth, invasion and evasion. Mol Metab.

[18] Warburg O (1956). On the origin of cancer cells. Science.

[19] House SW (1956). On respiratory impairment in cancer cells. Science.

[20] Lellahi SM (2018). The long noncoding RNA NEAT1 and nuclear paraspeckles are up-regulated by the transcription factor HSF1 in the heat shock response. J Biol Chem.

[21] Schopf FH, Biebl MM, Buchner J (2017). The HSP90 chaperone machinery. Nat Rev Mol Cell Biol.

[22] Iosefson O (2012). Reactivation of protein aggregates by mortalin and Tid1—the human mitochondrial Hsp70 chaperone system. Cell Stress Chaper.

[23] Kaufman DM (2017). Ageing and hypoxia cause protein aggregation in mitochondria. Cell Death Differ.

[24] Ruan L (2017). Cytosolic proteostasis through importing of misfolded proteins into mitochondria. Nature.

[25] Labbadia J (2017). Mitochondrial stress restores the heat shock response and prevents proteostasis collapse during aging. Cell Rep.

[26] Muftuoglu M, Mori MP, De Souza-Pinto NC (2014). Formation and repair of oxidative damage in the mitochondrial DNA. Mitochondrion.

[27] Sena LA, Chandel NS (2012). Physiological roles of mitochondrial reactive oxygen species. Mol Cell.

[28] Koritzinsky M (2013). Two phases of disulfide bond formation have differing requirements for oxygen. J Cell Biol.

[29] May D (2005). Ero1-L α plays a key role in a HIF-1-mediated pathway to improve disulfide bond formation and VEGF secretion under hypoxia: implication for cancer. Oncogene.

[30] Kueh HY, Niethammer P, Mitchison TJ (2013). Maintenance of mitochondrial oxygen homeostasis by cosubstrate compensation. Biophys J.

[31] Shpilka T, Haynes CM (2018). The mitochondrial UPR: mechanisms, physiological functions and implications in ageing. Nat Rev Mol Cell Biol.

[32] Quirós PM (2017). Multi-omics analysis identifies ATF4 as a key regulator of the mitochondrial stress response in mammals. J Cell Biol.

[33] Fiorese CJ (2016). The transcription factor ATF5 mediates a mammalian mitochondrial UPR. Curr Biol.

[34] Münch C (2018). The different axes of the mammalian mitochondrial unfolded protein response. BMC Biol.

[35] Vyas S, Zaganjor E, Haigis MC (2016). Mitochondria and cancer. Cell.

[36] Deng P, Haynes CM (2017). Mitochondrial dysfunction in cancer: potential roles of ATF5 and the mitochondrial UPR. In Semin Cancer Biol.

[37] Kenny TC, Germain D (2017). From discovery of the CHOP axis and targeting ClpP to the identification of additional axes of the UPRmt driven by the estrogen receptor and SIRT3. J Bioenerg Biomembr.

[38] Nargund AM (2012). Mitochondrial import efficiency of ATFS-1 regulates mitochondrial UPR activation. Science.

[39] Karpel-Massler G (2016). A synthetic cell-penetrating dominant-negative ATF5 peptide exerts anticancer activity against a broad spectrum of treatment-resistant cancers. Clin Cancer Res.

[40] Angelastro JM (2017). Targeting ATF5 in cancer. Trends Cancer.

[41] Sun X (2020). Dominant-negative ATF5 compromises cancer cell survival by targeting CEBPB and CEBPD. Mol Cancer Res.

[42] Ishihara S (2015). Activating transcription factor 5 enhances radioresistance and malignancy in cancer cells. Oncotarget.

[43] Wu CC (2016). The Apaf-1 apoptosome induces formation of caspase-9 homo-and heterodimers with distinct activities. Nat Commun.

[44] Gogada R (2013). Bim, a proapoptotic protein, up-regulated via transcription factor E2F1-dependent mechanism, functions as a prosurvival molecule in cancer. J Biol Chem.

[45] Gibson BA, Kraus WL (2012). New insights into the molecular and cellular functions of poly (ADP-ribose) and PARPs. Nat Rev Mol Cell Biol.

[46] Mohrin M (2015). A mitochondrial UPR-mediated metabolic checkpoint regulates hematopoietic stem cell aging. Science.

[47] Jin C (2016). Activation of IRE1α-XBP1 pathway induces cell proliferation and invasion in colorectal carcinoma. Biochem Biophys Res Commun.

[48] Gifford JB (2016). Expression of GRP78, master regulator of the unfolded protein response, increases chemoresistance in pancreatic ductal adenocarcinoma. Mol Cancer Ther.

[49] Yang D, Kim J (2019). Mitochondrial retrograde signalling and metabolic alterations in the tumour microenvironment. Cells.

[50] Wu CW (2005). Mitochondrial DNA mutations and mitochondrial DNA depletion in gastric cancer. Genes Chrom Cancer.

[51] Tseng LM (2006). Mitochondrial DNA mutations and mitochondrial DNA depletion in breast cancer. Genes Chrom Cancer.

[52] Lee HC, Wei YH (2009). Mitochondrial DNA instability and metabolic shift in human cancers. Int J Mol Sci.

[53] Lee HC (2014). Somatic alterations in mitochondrial DNA and mitochondrial dysfunction in gastric cancer progression. World J Gastroenterol.

[54] Lièvre A (2005). Clinical value of mitochondrial mutations in colorectal cancer. J Clin Oncol.

[55] Ishikawa K (2008). ROS-generating mitochondrial DNA mutations can regulate tumor cell metastasis. Science.

[56] Imanishi H (2011). Mitochondrial DNA mutations regulate metastasis of human breast cancer cells. PLoS ONE.

[57] Guo ZS (2017). Analysis of the mitochondrial 4977 bp deletion in patients with hepatocellular carcinoma. Balk J Med Genet.

[58] Máximo V (2001). Microsatellite instability, mitochondrial DNA large deletions, and mitochondrial DNA mutations in gastric carcinoma. Genes Chrom Cancer.

[59] Juan W, Lü YY (2009). Mitochondrial DNA 4977-bp deletion correlated with reactive oxygen species production and manganese superoxide dismutase expression in gastric tumor cells. Chin Med J.

[60] Tseng LM (2009). Association between mitochondrial DNA 4,977 bp deletion and NAD (P) H: quinone oxidoreductase 1 C609T polymorphism in human breast tissues. Oncol Rep.

[61] Santidrian AF (2013). Mitochondrial complex I activity and NAD+/NADH balance regulate breast cancer progression. J Clin Invest.

[62] Petros JA (2005). mtDNA mutations increase tumorigenicity in prostate cancer. Proc Natl Acad Sci USA.

[63] Porporato PE (2018). Mitochondrial metabolism and cancer. Cell Res.

[64] McMahon S, LaFramboise T (2014). Mutational patterns in the breast cancer mitochondrial genome, with clinical correlates. Carcinogenesis.

[65] Yuan Y (2015). Nonsense and missense mutation of mitochondrial ND6 gene promotes cell migration and invasion in human lung adenocarcinoma. BMC Cancer.

[66] Lehtonen HJ (2006). Increased risk of cancer in patients with fumarate hydratase germline mutation. J Med Genet.

[67] Gault MD (2018). Germline SDHA mutations in children and adults with cancer. Mol Case Stud.

[68] Cairns RA, Mak TW (2013). Oncogenic isocitrate dehydrogenase mutations: mechanisms, models, and clinical opportunities. Cancer Discov.

[69] Alhazzazi TY (2011). Sirtuin-3 (SIRT3), a novel potential therapeutic target for oral cancer. Cancer.

[70] Mahjabeen I, Kayani MA (2016). Loss of mitochondrial tumor suppressor genes expression is associated with unfavorable clinical outcome in head and neck squamous cell carcinoma: Data from retrospective study. PLoS ONE.

[71] Vara-Perez M, Felipe-Abrio B, Agostinis P (2019). Mitophagy in cancer: a tale of adaptation. Cells.

[72] Jazwinski SM (2013). The retrograde response: when mitochondrial quality control is not enough. Biochim Biophy Acta, Mol Cell Res.

[73] Nguyen T, Nioi P, Pickett CB (2009). The Nrf2-antioxidant response element signaling pathway and its activation by oxidative stress. J Biol Chem.

[74] Acín-Pérez R (2014). ROS-triggered phosphorylation of complex II by Fgr kinase regulates cellular adaptation to fuel use. Cell Metab.

[75] Chae S (2013). A systems approach for decoding mitochondrial retrograde signaling pathways. Sci Signal.

[76] Wang CY (1999). Control of inducible chemoresistance: enhanced anti-tumor therapy through increased apoptosis by inhibition of NF-κB. Nat Med.

[77] Biswas G (2003). Mitochondria to nucleus stress signaling: a distinctive mechanism of NFκB/Rel activation through calcineurin-mediated inactivation of IκBβ. J Cell Biol.

[78] Papa L, Germain D (2011). Estrogen receptor mediates a distinct mitochondrial unfolded protein response. J Cell Sci.

[79] Papa L, Germain D (2014). SirT3 regulates the mitochondrial unfolded protein response. Mol Cell Biol.

[80] Wek RC, Jiang HY, Anthony TG (2006). Coping with stress: eIF2 kinases and translational control. Biochem Soc Trans.

[81] Ye J (2010). The GCN2-ATF4 pathway is critical for tumour cell survival and proliferation in response to nutrient deprivation. EMBO J.

[82] Dey S (2015). ATF4-dependent induction of heme oxygenase 1 prevents anoikis and promotes metastasis. J Clin Invest.

[83] Angelastro JM (2017). Targeting ATF5 in cancer. Trend Cancer.

[84] Deng P, Haynes CM (2018). Mitochondrial dysfunction in cancer: potential roles of ATF5 and the mitochondrial UPR. Semin Cancer Biol.

[85] Ahmed MW (2019). Relationship of single nucleotide polymorphisms and haplotype interaction of mitochondrial unfolded protein response pathway genes with head and neck cancer. Future Oncol.

[86] Zhao R, et al. Dual targeting of mitochondrial unfolded protein response and BCL2 in acute myeloid leukemia. Blood. 2019; 2569.

[87] O'Malley J (2020). Mitochondrial stress response and cancer. Trends Cancer.

[88] Daverey A (2019). Depletion of mitochondrial protease OMA1 alters proliferative properties and promotes metastatic growth of breast cancer cells. Sci Rep.

[89] Cole A (2015). Inhibition of the mitochondrial protease ClpP as a therapeutic strategy for human acute myeloid leukemia. Cancer Cell.

[90] Seo JH (2016). The mitochondrial unfoldase-peptidase complex ClpXP controls bioenergetics stress and metastasis. PLoS Biol.

[91] Chen FM (2020). Activation of mitochondrial unfolded protein response is associated with Her2-overexpression breast cancer. Breast Cancer Res Treat.

[92] Arnold RS (2015). Bone metastasis in prostate cancer: recurring mitochondrial DNA mutation reveals selective pressure exerted by the bone microenvironment. Bone.

[93] Riar AK (2015). Mitochondrial dysfunction in breast cancer. Res Rep Biol.

[94] Hu YL (2012). Hypoxia-induced autophagy promotes tumor cell survival and adaptation to antiangiogenic treatment in glioblastoma. Cancer Res.

[95] Kenny TC (2019). Mitohormesis primes tumor invasion and metastasis. Cell Rep.

[96] Lin S (2019). Fascin controls metastatic colonization and mitochondrial oxidative phosphorylation by remodeling mitochondrial actin filaments. Cell Rep.

[97] Sumpter R (2016). Fanconi anemia proteins function in mitophagy and immunity. Cell.

[98] Martinez-Outschoorn UE (2012). BRCA1 mutations drive oxidative stress and glycolysis in the tumor microenvironment: implications for breast cancer prevention with antioxidant therapies. Cell Cycle.

[99] Agnihotri S (2016). PINK1 is a negative regulator of growth and the Warburg effect in glioblastoma. Cancer Res.

[100] Chourasia AH (2015). Mitophagy defects arising from Bnip3 loss promote mammary tumor progression to metastasis. EMBO Rep.

[101] Niu Y (2019). RNA N6-methyladenosine demethylase FTO promotes breast tumor progression through inhibiting BNIP3. Mol Cancer.

[102] Patel J (2020). DNA damage and mitochondria in cancer and aging. Carcinogenesis.

[103] van Gisbergen MW (2020). Mitochondrial dysfunction inhibits hypoxia-induced HIF-1α stabilization and expression of its downstream targets. Front Oncol.

[104] van Kuijk SJ (2016). Prognostic significance of carbonic anhydrase IX expression in cancer patients: a meta-analysis. Front Oncol.

[105] Finley LW (2011). SIRT3 opposes reprogramming of cancer cell metabolism through HIF1α destabilization. Cancer Cell.

[106] Li C (2018). PINK1 and PARK2 suppress pancreatic tumorigenesis through control of mitochondrial iron-mediated immunometabolism. Dev Cell.

[107] Kim S, Sieburth D (2018). Sphingosine kinase activates the mitochondrial unfolded protein response and is targeted to mitochondria by stress. Cell Rep.

[108] Macleod KF (2020). Mitophagy and mitochondrial dysfunction in cancer. Ann Rev Cancer Biol.

[109] Yu C (2017). Mitochondrial calcium uniporter as a target of microRNA-340 and promoter of metastasis via enhancing the Warburg effect. Oncotarget.

[110] Gogvadze V, Zhivotovsky B, Orrenius S (2010). The Warburg effect and mitochondrial stability in cancer cells. Mol Aspects Med.

[111] Deng X (2020). Pyruvate dehydrogenase kinase 1 interferes with glucose metabolism reprogramming and mitochondrial quality control to aggravate stress damage in cancer. J Cancer.

[112] Sun X (2018). Mitochondrial fission promotes cell migration by Ca2+/CaMKII/ERK/FAK pathway in hepatocellular carcinoma. Liver Int.

[113] Kim HJ, Maiti P, Barrientos A (2017). Mitochondrial ribosomes in cancer. Semin Cancer Biol.

[114] Lee JJ (2018). Inhibition of epithelial cell migration and Src/FAK signaling by SIRT3. Proc Natl Acad Sci USA.

[115] Sun X (2018). Increased mtDNA copy number promotes cancer progression by enhancing mitochondrial oxidative phosphorylation in microsatellite-stable colorectal cancer. Signal Transduct Target Ther.

[116] Lee WR (2017). Transcriptomic analysis of mitochondrial TFAM depletion changing cell morphology and proliferation. Sci Rep.

[117] Isaacs JS (2005). HIF overexpression correlates with biallelic loss of fumarate hydratase in renal cancer: novel role of fumarate in regulation of HIF stability. Cancer Cell.

[118] Selak MA (2005). Succinate links TCA cycle dysfunction to oncogenesis by inhibiting HIF-α prolyl hydroxylase. Cancer Cell.

[119] Latini A (2005). Mitochondrial energy metabolism is markedly impaired by D-2-hydroxyglutaric acid in rat tissues. Mol Genet Metab.

[120] Dempster JM, et al. Extracting biological insights from the project achilles genome-scale CRISPR screens in cancer cell lines. BioRxiv. 2019; 720243.

[121] Knott ME (2016). Circulating fibroblast growth factor 21 (Fgf21) as diagnostic and prognostic biomarker in renal cancer. J Mol Biomark Diagn.

[122] Li C (2016). Growth differentiation factor 15 is a promising diagnostic and prognostic biomarker in colorectal cancer. J Cell Mol Med.

[123] Tian Y (2016). Mitochondrial stress induces chromatin reorganization to promote longevity and UPRmt. Cell.

[124] Merkwirth C (2016). Two conserved histone demethylases regulate mitochondrial stress-induced longevity. Cell.

[125] Aldridge JE, Horibe T (2007). Hoogenraad NJ Discovery of genes activated by the mitochondrial unfolded protein response (mtUPR) and cognate promoter elements. PLoS ONE.

[126] Cruz-Bermúdez A (2015). Enhanced tumorigenicity by mitochondrial DNA mild mutations. Oncotarget.

[127] Gitschlag BL (2016). Homeostatic responses regulate selfish mitochondrial genome dynamics in *C. elegans*. Cell Metab.

[128] Kenny TC, Germain D (2017). mtDNA, metastasis, and the mitochondrial unfolded protein response (UPRmt). Front Cell Dev Biol.

[129] Wang H (2012). An omics strategy for discovering pulmonary biomarkers potentially relevant to the evaluation of tobacco products. Biomark Med.

[130] Sharma A (2019). Hypoxia-targeted drug delivery. Chem Soc Rev.

[131] Birle DC, Hedley DW (2007). Suppression of the hypoxia-inducible factor-1 response in cervical carcinoma xenografts by proteasome inhibitors. Cancer Res.

[132] Roccaro AM (2006). Bortezomib mediates antiangiogenesis in multiple myeloma via direct and indirect effects on endothelial cells. Cancer Res.

[133] Pore N (2006). HIV protease inhibitors decrease VEGF/HIF-1α expression and angiogenesis in glioblastoma cells. Neoplasia.

[134] Pore N (2006). Nelfinavir down-regulates hypoxia-inducible factor 1α and VEGF expression and increases tumor oxygenation: implications for radiotherapy. Cancer Res.

[135] Isaacs JS (2002). Hsp90 regulates a von Hippel Lindau-independent hypoxia-inducible factor-1α-degradative pathway. J Biol Chem.

[136] Siegelin MD (2011). Exploiting the mitochondrial unfolded protein response for cancer therapy in mice and human cells. J Clin Invest.

[137] Kang BH (2009). Combinatorial drug design targeting multiple cancer signaling networks controlled by mitochondrial Hsp90. J Clin Invest.

[138] Papa L, Manfredi G, Germain D (2014). SOD1, an unexpected novel target for cancer therapy. Genes Cancer.

[139] Li S (2018). Disrupting SOD1 activity inhibits cell growth and enhances lipid accumulation in nasopharyngeal carcinoma. Cell Commun Signal.

[140] Dong X (2016). The rational design of specific SOD1 inhibitors via copper coordination and their application in ROS signaling research. Chem Sci.

[141] Glasauer A (2014). Targeting SOD1 reduces experimental non–small-cell lung cancer. J Clin Invest.

[142] Leone RD (2019). Glutamine blockade induces divergent metabolic programs to overcome tumor immune evasion. Science.

[143] Scott K (2016). Bortezomib for the treatment of multiple myeloma. Cochrane Database Syst Rev.

[144] Zang Y (2015). Carfilzomib and ONX 0912 inhibit cell survival and tumor growth of head and neck cancer and their activities are enhanced by suppression of Mcl-1 or autophagy. Clin Cancer Res.

[145] Skrott Z (2017). Alcohol-abuse drug disulfiram targets cancer via p97 segregase adaptor NPL4. Nature.

[146] Xu B (2016). Celecoxib induces apoptosis but up-regulates VEGF via endoplasmic reticulum stress in human colorectal cancer in vitro and in vivo. Cancer Chemother Pharmacol.

[147] Lin YC (2014). Metformin sensitizes anticancer effect of dasatinib in head and neck squamous cell carcinoma cells through AMPK-dependent ER stress. Oncotarget.

[148] Wang X (2018). Dasatinib promotes TRAImediated apoptosis by upregulating CHOP-dependent death receptor 5 in gastric cancer. FEBS Open Biol.

[149] Berridge MV, Crasso C, Neuzil J (2018). Mitochondrial genome transfer to tumor cells breaks the rules and establishes a new precedent in cancer biology. Mol Cell Oncol.

[150] Zhao Z (2020). Improvement of cognitive and motor performance with mitotherapy in aged mice. Int J Biol Sci.

[151] Pasquier J (2013). Preferential transfer of mitochondria from endothelial to cancer cells through tunneling nanotubes modulates chemoresistance. J Transl Med.

[152] Chang JC (2019). Mitochondrial transplantation regulates antitumour activity, chemoresistance and mitochondrial dynamics in breast cancer. J Exp Clin Cancer Res.

[153] Burt R (2019). Activated stromal cells transfer mitochondria to rescue acute lymphoblastic leukemia cells from oxidative stress. Blood.

[154] Goard CA, Schimmer AD (2014). Mitochondrial matrix proteases as novel therapeutic targets in malignancy. Oncogene.

[155] Ishizawa J (2019). Mitochondrial ClpP-mediated proteolysis induces selective cancer cell lethality. Cancer Cell.

[156] Mirali S (2020). The mitochondrial peptidase, neurolysin, regulates respiratory chain supercomplex formation and is necessary for AML viability. Sci Transl Med.

[157] Ishikawa K (2008). Reversible regulation of metastasis by ROS-generating mtDNA mutations. Mitochondrion.

